# Turbidity and streamflow as real-time indicators of microbial risk for aquatic recreators

**DOI:** 10.1007/s10661-026-15370-6

**Published:** 2026-04-28

**Authors:** Elliot S. Anderson, Christopher S. Jones, Claire E. Hruby

**Affiliations:** 1 Iowa Geological Survey, University of Iowa, Iowa City, IA USA; 2 IIHR—Hydroscience & Engineering, University of Iowa, Iowa City, IA USA; 3 Department of Environmental Science & Sustainability, Drake University, Des Moines, IA USA

**Keywords:** *E. coli*, Recreation, Turbidity, Waterborne, Streamflow, Surrogacy

## Abstract

Aquatic recreation is a popular pastime, but recreators often incur the risk of acquiring illnesses through exposure to waterborne pathogens. Several standards advise stakeholders on a waterbody’s recreational suitability using indicator bacteria concentrations. However, quantifying indicator organisms often takes several hours or days, resulting in a lack of real-time information. Microbe concentrations can also fluctuate dramatically over short timeframes, making it difficult to systematically assess and communicate risk using discrete samples. This study investigated turbidity and streamflow as real-time predictors of *Escherichia coli* (*E. coli*) in two major Iowa waterways (the Des Moines and Raccoon Rivers). A large historical dataset (*n* > 4000 at both sites) was leveraged to construct logistic models that predicted the probability of *E. coli* exceeding two recreational water quality standards (235 and 2880 organisms/100 mL). High-resolution turbidity measurements were collected to implement these models and comprehensively estimate long-term exceedance levels of the standards. Models were created at each site that used turbidity as the sole predictor—streamflow proved unnecessary. While these models were viable, their performance was relatively weak (pseudo *R*^2^ between 0.19 and 0.38, sensitivities between 6.8 and 68%), suggesting that turbidity and streamflow alone are likely insufficient for producing real-time predictions of risk at accuracy levels acceptable to stakeholders. Still, there may be utility in deploying similar models to describe a waterway’s propensity to exceed *E. coli* thresholds over the course of several months or years and to prioritize improvement efforts.

**Supplementary information:**

The online version contains supplementary material available at 10.1007/s10661-026-15370-6.

## Introduction

Aquatic recreation is among the most popular pastimes in the United States (US) and worldwide (Pitt, 1989; WHO, 2003). In addition to the pleasure it provides, aquatic recreation has been shown to instill numerous physical (Godbey, 2009; McDougall et al., 2022) and mental health benefits (Lackey et al., 2021; Overbury et al., 2023; Zhang et al., 2021). While aquatic recreation does not have a universal definition, many activities are commonly considered to fall within its scope, including swimming, fishing, paddling, kayaking, boating, and playing watersports. Many communities are actively seeking to increase opportunities for aquatic recreation to spur economic development and population growth (Tribe, 2020).

While benefits from recreation abound, several risks arise anytime individuals partake in these activities (Dorevitch et al., 2012; Pakasi, 2018). One of the biggest risks associated with recreating in surface waters (e.g., rivers, streams, and lakes) is infection from pathogen exposure (Russo et al., 2020). Pathogens are infectious microorganisms that can cause disease. While numerous microorganisms, including bacteria, viruses, and protozoa, reside in all environmental waters, a small subset of these that can cause negative human health implications (i.e., pathogens) may also be present. In instances where these pathogens contaminate surface waters, recreators are at risk of acquiring illnesses or infections.

Waterborne exposure commonly occurs via incidental ingestion but can also take place through direct contact, where pathogens enter a body through cuts or abrasions on the skin or various orifices (Gerba, 2009). Therefore, recreational activities that result in full-body immersion or prolonged contact with surface waters (i.e., primary contact) are generally viewed as riskier than activities where water contact is minimal (i.e., secondary contact) (Boehm & Soller, 2012). Waterborne pathogen transmission often causes minor gastrointestinal and skin infections, but in certain circumstances, serious diseases can result (Adhikary et al., 2022). Some of the more severe diseases arising from exposure during aquatic recreation include campylobacteriosis (Pitkänen, 2013), cryptosporidiosis (Gallaher et al., 1989), and giardiasis (Hamilton et al., 2018). Symptoms of such diseases often include stomach pain, diarrhea, cramping, nausea, and fever.

Since a wide variety of microorganisms can be detrimental to human health, determining their individual presence in a waterbody is usually impractical. Rather, the most common strategy for determining waterborne pathogen levels is quantifying the abundance of an indicator microbe species that reflects overall pathogen presence associated with symptomatic illnesses (Saxena et al., 2015). These indicator pathogens may themselves not be harmful, but high concentrations have been shown to correlate with microorganisms of concern, such as *Campylobacter*, *Cryptosporidium*, and *Giardia,* which trigger the aforementioned diseases upon infection (Korajkic et al., 2018). Over the past several decades, *Escherichia coli* (*E. coli*) have become widely used indicator organisms (Ishii & Sadowsky, 2008). They are relatively inexpensive to measure (Francy & Darner, 2000), and their presence has been linked to increased infection rates (Leclerc et al., 2002; Nwabor et al., 2016). Consequently, *E. coli* are a staple of most modern sampling programs and among the most frequently measured water quality parameters in the US (Cho et al., 2020).

Several standards have been developed that aim to describe a waterbody’s recreational risk level based on *E. coli* measurements. Two of the most widely used standards were constructed by the US Environmental Protection Agency (EPA) and correspond to activities resulting in primary contact (Class A1) and secondary contact (Class A2) with surface waters (Fujioka et al., 2015). The Class A1 standard for a waterbody is violated when a single sample exceeds an *E. coli* concentration of 235 organisms/100 mL or the geometric mean of five weekly samples exceeds 126 organisms/100 mL. When this occurs, local agencies will issue advisories recommending against activities involving full-body immersion (e.g., swimming). The Class A2 standard is violated when *E. coli* exceeds thresholds of 2880 (single sample maximum) or 630 (five-week geometric mean) organisms/100 mL. Here, agencies will recommend against most aquatic recreation (Wade et al., 2003).

While utilizing *E. coli* standards to advise the public has been beneficial (Wade et al., 2003), this approach has the limitation of being unable to provide real-time information (Rossi et al., 2020). Most of the popular *E. coli* quantification methods require a 24-h incubation period. Thus, *E. coli* levels in a waterbody at any moment in time it is being used by recreators are often a matter of conjecture. Furthermore, *E. coli* concentrations can be highly dynamic—routinely spanning several orders of magnitude following point-source discharges (Garcia-Armisen & Servais, 2007) or rainfall events (Harmel et al., 2010) and containing little autocorrelation (Anderson & Schilling, 2024). The amount of waterborne *E. coli* present at any given time can vastly differ from long-term averages (McCarthy et al., 2008). These factors make it difficult to infer real-time risk levels of pathogen exposure (Ramírez-Castillo et al., 2015).

One common method for quantifying analytes that elude real-time measurement is the use of surrogacy models. These models traditionally input “easy-to-measure” parameters that can be monitored continuously in situ into regression models that predict concentrations of “hard-to-measure” parameters that require laboratory analysis for direct quantification (Robertson et al., 2018). Surrogacy models are typically site-specific and rely on historical water quality data to estimate model parameters (Castrillo & García, 2020). These models have been utilized to great effect for various problematic analytes, including sediment (Gray et al., 2010), phosphorus (Viviano et al., 2014), and salinity (Sreekanth & Datta, 2010). Along with providing real-time estimates, they can also enable high-resolution descriptions of analytes whose records were previously confined to discrete sampling (Gray et al., 2010).

Two of the most utilized surrogates are streamflow and turbidity (Robertson et al., 2018; Steffy & Shank, 2018). Streamflow is widely measured due to its significant implications concerning water availability and flood risk. Streamflow is often related to *E. coli* as precipitation that raises flow levels can also transport bacteria into waterways via runoff or subsurface drainage networks (Hruby et al., 2016; Soupir et al., 2006) or resuspension from bottom sediments (Pandey & Soupir, 2013). Turbidity, which is a qualitative measure of water clarity, is another widely used parameter due to its associations with sediment transport and ecological health (Lunt & Smee, 2020), and in situ turbidity datasets are now routinely collected using turbidimeters (Omar & MatJafri, 2009). Higher turbidity values are also linked with pathogen presence (Hamilton & Luffman, 2009). Opaque materials, including sediment and organic matter, that decrease water clarity often contain pathogens (Dorner et al., 2007).

While surrogacy models have been successfully deployed for a variety of purposes, efforts to develop surrogacy models describing microorganism behavior have generally proven less successful (Ramírez-Castillo et al., 2015; Rossi et al., 2020; Sinclair et al., 2012). In many cases, the potential range of *E. coli* values is vast and hard to capture using traditional regression techniques (Dada, 2019; Sokolova et al., 2022). In addition, many waterbodies with high levels of indicator bacteria often receive these microorganisms from disparate sources, each with varying degrees of connectivity to hydrology (Korajkic et al., 2018). The mathematical forms of microorganism-based surrogacy models can be hard to generalize, as bacteria transport pathways are often unique to local conditions (McKee & Cruz, 2021).

Still, the links between indicator bacteria, hydrology, and water clarity are well-documented (Dorner et al., 2006; Vermeulen et al., 2015; Wu et al., 2009). While surrogacy models have struggled to estimate *E. coli* concentrations, we wanted to investigate their use in relation to the EPA *E. coli* standards. Such a method involves reframing traditional surrogacy models in a classification context. Whereas the traditional approach uses regression models to predict *E. coli* concentrations, a revised approach would use classification models to predict whether recreational thresholds have been exceeded.

In this study, we explore the ability of surrogacy models to predict exceedance of recreational *E. coli* standards in two major rivers in central Iowa. Our objectives were to (1) determine the viability of turbidity and streamflow as surrogates in predicting whether *E. coli* concentrations exceed the Class A1 and A2 EPA standards, (2) collect in situ data to implement these models, and (3) use the models to estimate the percentage of time these rivers have historically exceeded the *E. coli* standards. We specifically selected locations that (1) were commonly used for recreation and (2) have substantial historical water quality data. The hope was that these sites would provide a “best case” scenario for model construction based on data availability and recreational usage.

## Methods

### Site description and setting

**Fig. 1:**
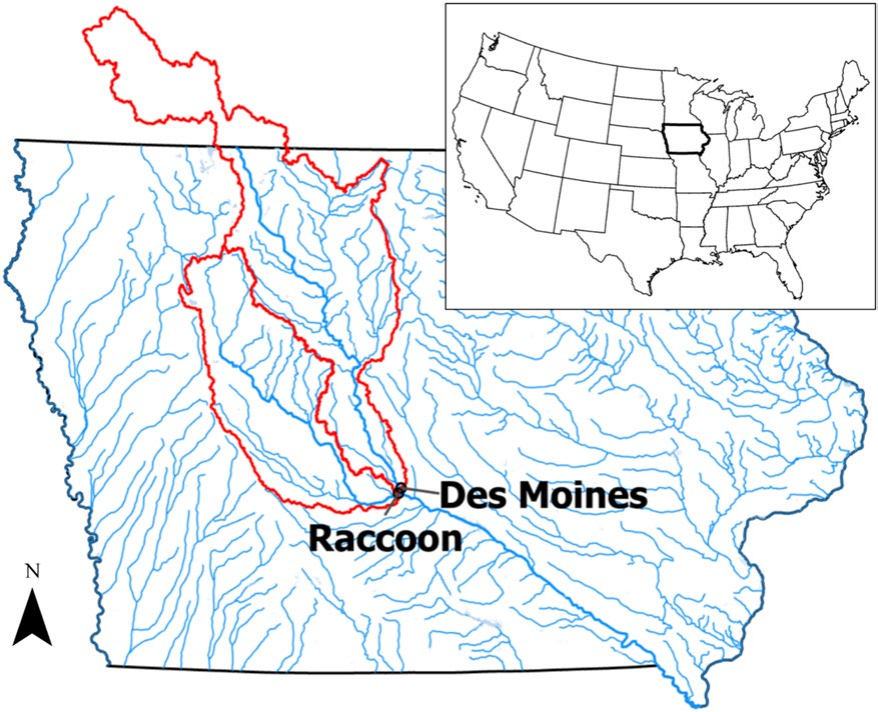
Map of site locations and their corresponding watersheds. The main stems of the Des Moines and Raccoon Rivers are emboldened

This study centered on two sites located along major rivers in Des Moines, IA (Table 1). Figure 1 displays these two sites and their corresponding watersheds. Des Moines is the most populous city and capital of the State of Iowa, with approximately 600,000 people living within its urban area. Waterbodies near Des Moines are part of the larger HUC6 watershed (071000), which encompasses the Des Moines River and flows from its headwaters in Minnesota to its confluence with the Mississippi River near Keokuk, IA. The Raccoon River, the largest tributary of the Des Moines River, discharges into the main stem in the heart of the city of Des Moines.

**Table 1:** Summary of site information and DMWW water quality data

Short name	Full name	USGSid	IWQISid	Area (km^2^)	Lat	Long	*E. coli* samples	% Above 235	% Above 2880	Turb samples
Des Moines	Des Moines River at 2nd Avenue at Des Moines, IA	05482000	WQS0092	16,174	41.6125	−93.620833	4188	16.6%	2.1%	4323
Raccoon	Raccoon River at Fleur Drive at Des Moines, IA	05484900	WQS0098	9389	41.581667	−93.642778	4175	35.3%	9.3%	4362

This confluence has historically been the center of significant economic activity and is located in the city’s downtown area. Large urban and suburban communities surround both rivers within the Des Moines metropolitan area, with the main river branches and their tributaries increasingly serving as recreational destinations from May to September. Further upstream, the Des Moines and Raccoon Rivers watersheds are predominantly rural, with conventional row crop agriculture dominating land use outside the Des Moines metropolitan area. Both rivers are perennial, registering flow values near 3 m^3^/s even under the driest conditions. Stakeholders closely monitor local hydrologic conditions, as streamflow levels can impact the rivers’ water quality and recreational suitability.

These rivers are also the primary drinking water source for the residents of Des Moines. Des Moines Water Works (DMWW) is the public utility that collects, treats, and distributes drinking water to stakeholders in the area. DMWW operates and continuously monitors water intakes along the Des Moines and Raccoon Rivers. Water from these locations is tested within on-site laboratory facilities for numerous analytes pertinent to drinking water contamination, including *E. coli* and turbidity. Streamflow is monitored continually via co-located US Geological Survey (USGS) stream gauges at both sites.

The first site, hereafter referred to as Des Moines, is located along the main stem of the Des Moines River, 4.3 km above its confluence with the Raccoon (Fig. 1). It corresponds to DMWW’s Des Moines River intake and USGS gauge #05482000 (Table 1). This site drains an area of 16,100 km^2^, which lies entirely within the Des Moines Lobe landform region. While the site is located in an urban environment, the larger watershed is dominated by agricultural landscapes.

Saylorville Lake, a large flood-mitigation reservoir, lies along the Des Moines River 9.7 km upstream. This reservoir stores water from over 95% of the site’s tributary area, considerably affecting its hydrology and water quality. Streamflow levels often reflect the decisions made by reservoir operators rather than natural hydrologic processes. Impounding water within the reservoir leads to large amounts of sedimentation and alters the fate of waterborne pathogens (Brookes et al., 2004). Many microorganisms settle or perish in these stagnant systems, and average residence times within Saylorville Lake typically span five to 30 days (Anderson & Schilling, 2025). It is thus common for turbidity and *E. coli* levels directly downstream of Saylorville Lake to be lower than most riverine locales in the State (Lutz et al., 1997). Nearby locations also influence water quality, as several tributaries downstream of Saylorville Lake drain urban areas to the DMWW site. While flows from these tributaries are dwarfed by those from Saylorville during normal hydrologic conditions, several contain notable point-source discharges that can impact pathogen concentrations. The most notable are wastewater treatment plants that have historically struggled with indicator bacteria in their effluent, but microbes have been traced to a variety of local sources (Burch et al., 2024).

The second site, hereafter referred to as Raccoon, is located along the Raccoon River, 3.9 km above its confluence with the Des Moines (Fig. 1). It corresponds to DMWW’s Raccoon River intake and USGS gauge #05484900 (Table 1). This site’s watershed area is 9400 km^2^ and is split between the Des Moines Lobe and Southern Iowa Drift Plain landform regions. Agricultural landscapes similarly dominate the watershed area. In contrast to the Des Moines site, the river here is more free-flowing. Several low-head dams are located along the Raccoon River, but no major flood impoundments exist. Consequently, the location’s water quality is more closely linked to hydrologic processes than the Des Moines site. Both the upstream agricultural landscape and nearby urban and suburban areas influence the site’s *E. coli* levels (Schilling et al., 2009).

### Water quality data

Historical *E. coli* and turbidity data were retrieved for both sites. DMWW collected these water quality datasets as part of routine monitoring at their water intakes. DMWW measures *E. coli* and turbidity at both sites every weekday (excluding major holidays). Collection times vary but are usually around 6:00 am. While sampling records extend back several decades, only data from 2006 to the present are readily available due to DMWW’s database structure. Data from 2006 to 2023 were made available for the authors by DMWW operators upon request.

*E. coli* was measured using Quanti-Tray analytical equipment and Colilert and Colisure tests, which mix reagents with sample water (Fricker et al., 1997). Reagents are added to the collected water then poured into the Quanti-Tray devices that contain numerous individual water wells. The devices are then sealed and incubated, and *E. coli* concentrations are inferred—i.e., the most probable number (MPN) is obtained—by counting the number of wells that fluoresce. Sampling procedures remained unchanged throughout the timeframe of this study’s dataset (2006–2023), and all *E. coli* results were reported using units of MPN/100 mL. In rare instances (< 1% of samples), analytical procedures resulted in censored *E. coli* values. This occurs when all or none of the individual Quanti-Tray wells fluoresce. Censored values can result in either a maximum (when all wells fluoresce) or minimum (when no wells fluoresce) bound on the result.

Turbidity was measured using Standard Method 2130-B, where the intensity of light scattered within a sample is quantified and compared to reference conditions (Davies‐Colley & Smith, 2001). All units were reported using nephelometric turbidity units (NTU), and no turbidity values were censored. All analytical procedures utilized by the DMWW for water quality monitoring conform to EPA-approved protocols for water treatment plants.

**Fig. 2:**
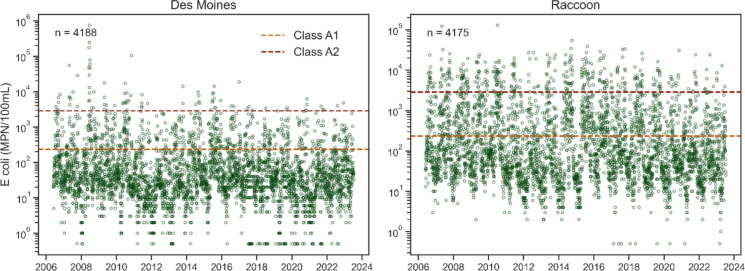
Historical DMWW *E. coli* data at the Des Moines (left) and Raccoon (right) sites. Horizontal lines are 235 (Class A1) and 2880 (Class A2) *E. coli* standards

From the 2006–2023 period, 4188 and 4175 *E. coli* measurements were taken at the Des Moines and Raccoon sites, respectively (Fig. 2). Over this timeframe, 16.6% of samples at Des Moines exceeded 235 MPN/100 mL (Class A1 standard), and 2.1% were above 2880 MPN/100 mL (Class A2 standard). At the Raccoon, 35.3% and 9.3% of samples exceeded these respective thresholds (Table 1). DMWW collected similar numbers of coincident turbidity samples.

Concurrent flow values were determined using daily mean streamflows at the co-located USGS gauges. Standard USGS protocols utilizing stage-discharge rating curves to measure streamflow have been deployed at both sites over several decades. USGS methods also involve retroactively estimating flow during instances of equipment failure (Hirsch & Costa, 2004), and thus, there were no missing streamflow values at either site during our analysis period. An extensive range of flow conditions was present in the DMWW datasets. Daily sampling over a 15+-year period has resulted in nearly all hydrologic states being captured within the DMWW monitoring. To visualize the relationship between streamflow, *E. coli* loads, and the *E. coli* recreational thresholds, load duration curves were created for both sites (Supplemental Materials).

### Logistic model framework

This study’s modeling framework used logistic models, with turbidity and streamflow as input variables, to predict the exceedance of *E. coli*-based recreational standards. Logistic models (also known as logit models) predict the probability of an event occurring—in this instance, waterborne *E. coli* exceeding the recreational standard. In our setup, logistic models took the general form

$$P\left(X\right)=\frac{1}{1+{e}^{-({\beta }_{0}+{\beta }_{1}*\mathrm{ln}\left[Turb\right]+{\beta }_{2}*\mathrm{ln}\left[Flow\right])}}$$

where *P*(*X*) is the probability of exceeding an *E. coli* recreational standard, ln[Turb] is the log-transformed turbidity variable, ln[Flow] is the log-transformed streamflow variable, and *β*_0_, *β*_1_, and *β*_2_ are coefficients estimated through model construction.

Turbidity and streamflow values were log-transformed prior to model construction, as this helped normalize both datasets, which were heavily skewed in their nontransformed state. Positive skew has routinely been noted in turbidity and streamflow data in Iowa (Anderson et al., 2024; Blum et al., 2017; Granato et al., 2017), and transforming these variables prior to analysis is common practice (Crowder et al., 2007; Helsel & Hirsch, 1993; Hirsch et al., 2010; Moog et al., 1999).

**Fig. 3:**
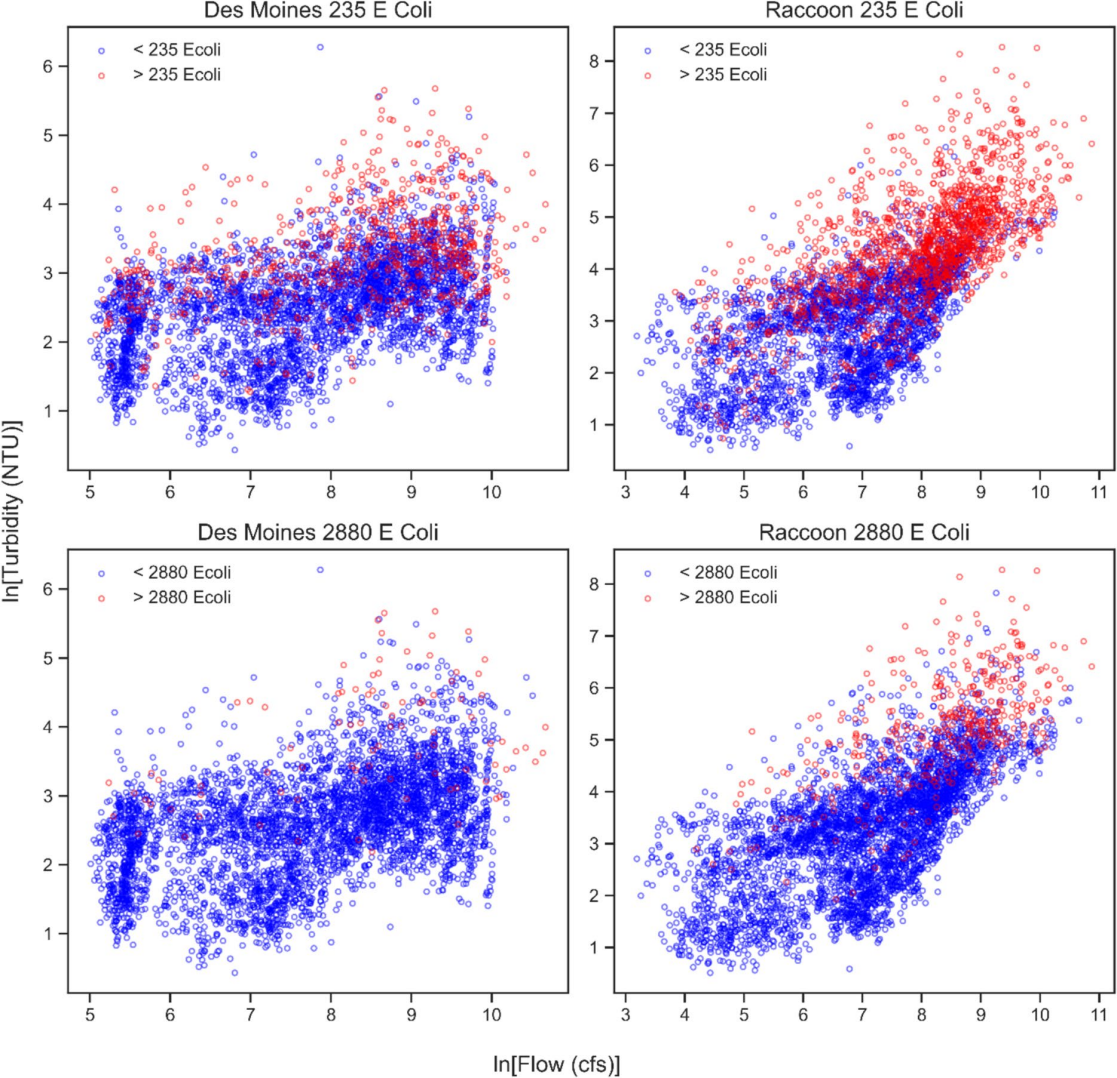
Classification plots for *E. coli* as a function of turbidity and streamflow. Samples are classified as measurements above (red) and below (blue) the *E. coli* standards

The probabilities these models produced helped define a decision boundary separating binary classes of points—those above the *E. coli* standard and those below. DMWW *E. coli* samples were classified based on whether they exceeded the 235 and 2880 recreational thresholds for *E. Coli* prescribed by the EPA, and Fig. 3 demonstrates this framework visually. For example, in the top left subplot of Fig. 3, a model would use flow and turbidity at the Des Moines site to predict the probability that concurrent *E. coli* is above 235 MPN/100 mL. A dividing line (i.e., a decision boundary) is then constructed. This is most often done where the exceedance probability is 0.5. On one side of the line, the model predicts *E. coli* will be above the 235 standard—on the other side, the model predicts *E. coli* will be below the standard.

Models were constructed at the sites for both the Class A1 and A2 standards. In each case, three models were explored: one using turbidity as the sole predictor, one using flow as the sole predictor, and one using a combination of both. The statsmodels Python package (version 0.13.5) was utilized for model construction, with maximum likelihood estimation used to optimize model parameters. Each model iteration evaluated statistical significance and goodness of fit. Goodness of fit was quantified using a metric known as pseudo *R*^2^, which is formally defined as

$$Pseudo {R}^{2}=1-\frac{\mathrm{ln}[\mathcal{L}\left({m}_{1}^{*}\right)]}{\mathrm{ln}[\mathcal{L}\left({m}_{0}^{*}\right)]}$$

where $\mathrm{ln}[\mathcal{L}\left({m}_{1}^{*}\right)]$ is the maximized value of the model’s log-likelihood function and $\mathrm{ln}[\mathcal{L}\left({m}_{0}^{*}\right)]$ is the maximized value of a log-likelihood function when only an intercept term is included in the model’s predictors. Much like the traditional coefficient of determination (*R*^2^), the pseudo *R*^2^ ranges from 0 to 1, with high values indicating better performance. The water quality data used to construct each model and the python scripts used to conduct our analysis have been included in Supplemental Materials.

**Fig. 4:**
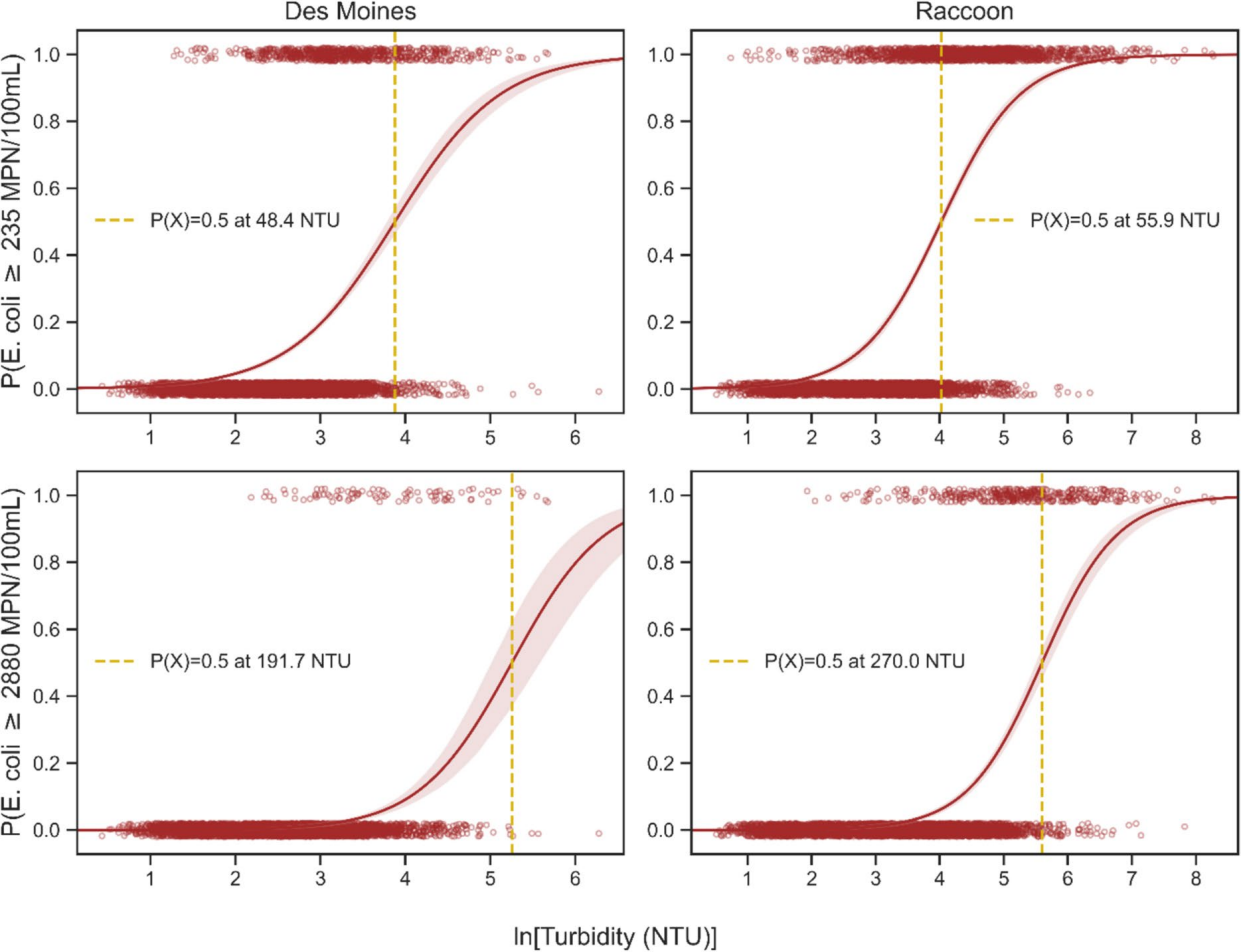
Logistic regression models using log-transformed turbidity to predict exceedance probability of the Class A1 (top) and Class A2 (bottom) *E. coli* standards. Vertical lines are the turbidity values at which the exceedance probability is 0.5, and shaded areas represent the 95% confidence interval

Final models were selected for each site and *E. coli* standard (Fig. 4). These four models were selected based on the statistical significance of the predictors and model performance. Once models were constructed, we classified their predictions using a probability of 0.5 as the decision boundary. As such, samples with a predicted probability greater than 0.5 were classified as being above the recreational standard. Confusion matrices were created to summarize model performance (Supplemental Materials), and the accuracy of each model was calculated to quantify the percentage of correct predictions.

### In situ turbidity measurements

To support the implementation of these models, turbidimeters were deployed to collect high-resolution in situ turbidity data. It should be noted that streamflow data continues to be collected at both sites by the USGS, so no additional effort was needed to gather high-resolution flow records. FTS DTS-12 turbidity sensors were used at both sites. These devices use nephelometry to provide continuous in situ turbidity measurement and have proved effective in several similar deployment scenarios (Loperfido et al., 2010; Mueller et al., 2013; Shinkareva et al., 2024; Snazelle, 2015).

The first sensor was deployed in September 2019 at the Des Moines site, and the second was installed at the Raccoon site in June 2020. The difference in start dates was due to several technical and logistical difficulties arising at the Raccoon site, which resulted in its deployment being delayed. Devices were programmed to measure turbidity every 15 min while deployed. Dataloggers and modems were connected to the sensors to digitally transfer and store their observations within the Iowa Water Quality Information System (IWQIS). IWQIS is a web-based informatics platform that communicates current and historical water quality data in Iowa to various stakeholders (Jones et al., 2018). The modems continually transmitted their measurements to IWQIS’s database and displayed them on the web platform, thereby providing real-time turbidity readings from these sensors.

**Fig. 5:**
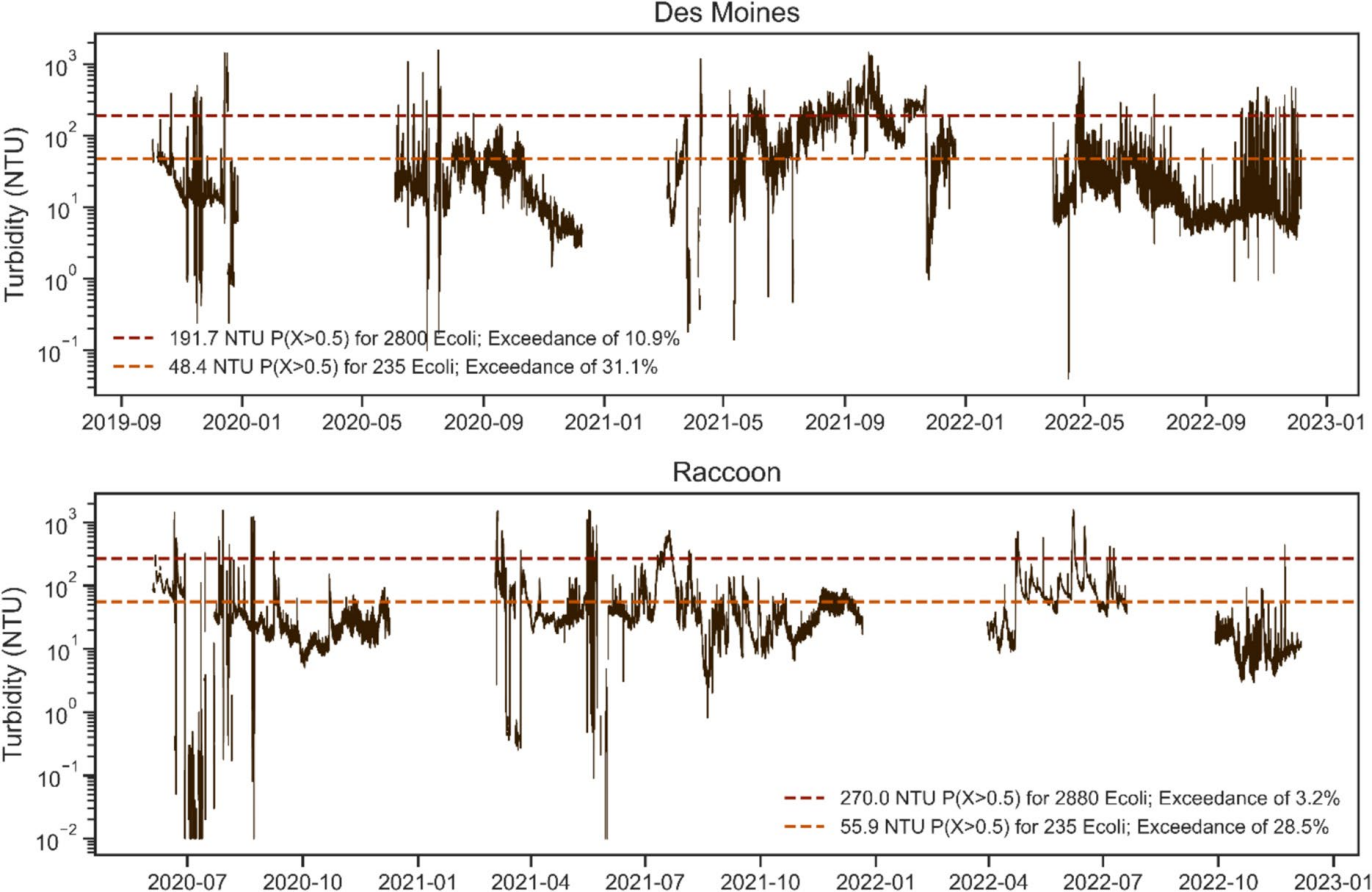
In situ turbidity measurements, along with turbidity thresholds corresponding to 50% exceedance of Class A1 and A2 standards

After their initial installation, the turbidimeters were retrieved for the winter months and redeployed the following spring. Each winter, the devices were temporarily returned to the manufacturer for recalibration and to ensure all equipment was operational. In situ data collection at both sites concluded in late 2022. Our specific deployment timelines were as follows: Des Moines (Sep–Dec 2019, May–Dec 2020, Mar–Dec 2021, and Mar–Dec 2022) and Raccoon (Jun–Dec 2020, Mar–Dec 2021, and May–Dec 2022). Minor issues with sensor fouling led to occasional gaps in turbidity data, but otherwise, continuous turbidity records were generated in the nonwinter months from 2019 to 2022 (Fig. 5). All observations were reported using units of NTU.

Upon final sensor retrieval, all in situ turbidity data were retrieved from the IWQIS webpage and postprocessed. The Des Moines and Raccoon sites correspond to IWQIS sites WQS0092 and WQS0098, respectively. These datasets were input into the final models to produce corresponding probability predictions. We thus used these in situ data to produce high-resolution records estimating the probability that the Des Moines and Raccoon Rivers had exceeded the Class A1 and A2 *E. coli* standards. We then averaged the probability output produced by each model to estimate the overall percentage of time that each river had exceeded the *E. coli* standard throughout the monitoring period. The resulting arithmetic means provided a convenient metric for gauging how often these rivers had exceeded each threshold over prolonged periods.

## Results

Each model iteration was successfully simulated, and complete model output details have been included in the Supplemental Materials.

### Logistic model selection and results

Three logistic models were created for both sites and *E. coli* standards. In each case, the model that used turbidity as the sole predictor was selected for implementation. Both turbidity and streamflow were always statistically significant (*p* < 0.001) in models that used them as individual predictors. However, turbidity-based models greatly outperformed their flow-based counterparts, with larger pseudo *R*^2^ values in every scenario (Table 2). Models that used both turbidity and flow as predictors did not improve upon the turbidity-only models. Flow was not statistically significant (*p* > 0.05) when included as a covariate alongside turbidity (Supplemental Materials), and pseudo *R*^2^ values are very similar (< 0.01 difference, Table 2). Ultimately, turbidity was the only variable needed to estimate *E. coli* threshold exceedance in our framework.

**Table 2:** Summary of model pseudo *R*^2^ values

Site	*E. coli* threshold	Turbidity	Flow	Turbidity & flow
Des Moines	235	0.19	0.03	0.19
	2880	0.23	0.03	0.23
Raccoon	235	0.35	0.15	0.35
	2880	0.38	0.14	0.39

Figure 4 contains the logistic regression curves for the four turbidity-based models, along with their underlying sample data. All curves were positive, where higher turbidity values yielded greater probabilities of exceedance. Model performance was better in the Raccoon (pseudo *R*^2^ values of 0.35 and 0.38) than the Des Moines (pseudo *R*^2^ values of 0.19 and 0.23). Pseudo *R*^2^ values were also slightly higher for the Class A2 standard than the Class A1 (difference of ~ 0.03).

Figure 4 also includes the turbidity thresholds corresponding to the 0.5 exceedance probability (vertical lines). At the Des Moines site, the threshold for the 235 *E. coli* standard is 48.4 NTU (Fig. 4, top left). Therefore, turbidity measurements above 48.4 NTU estimate that the water has a greater than 50% chance of exceeding the Class A1 recreational standard. This corresponding turbidity value at the Raccoon site is 55.9 NTU. The 0.5 exceedance thresholds were higher for the Class A2 standard, with values of 191.7 NTU for the Des Moines and 270.0 NTU for the Raccoon. This was expected, as the positive correlation between *E. coli* and turbidity suggests that greater turbidity is required to exceed a higher *E. coli* benchmark.

Confusion matrices were used to summarize model performance on the sample data when using a probability of 0.5 as the decision boundary (Supplemental Materials). These matrices count the number of true negatives (top left), true positives (bottom right), false negatives (bottom left), and false positives (top right). For example, a sample at the Des Moines site with a turbidity of 50 NTU but an *E. coli* value of 200 MPN/100 mL would be categorized as a false positive for the 235 *E. coli* model. This is because the turbidity observation was higher than the 48.4 NTU threshold—thereby predicting that the water had a greater than 50% chance of exceeding 235 MPN/100 mL, but the actual sample had *E. coli* levels below this standard.

A variety of metrics can be gleaned from these confusion matrices, but we aggregated the accuracy (% of correctly classified samples), sensitivity (% of correctly classified true positives), and specificity (% of correctly classified true negatives) to concisely summarize model performance (Table 3). Model accuracies varied between 82.3 and 97.9%, with the highest occurring in the Des Moines Class A2 model. Sensitivities demonstrated a considerable range. The Des Moines Class A2 model contained the lowest value (6.8%), while the Raccoon Class A1 sensitivity (67.6%) was easily the largest—other sensitivities were 21.7% and 34.7%. The Raccoon Class A1 specificity was 90.3%, while the other models ranged between 97.8 and 99.9%.

**Table 3:** Exceedance table for *E. coli* recreational standards of 235 and 2880. The sixth column indicates the turbidity corresponding to the 50% likelihood that *E. coli* levels will exceed the standards. Thus, when turbidity is above this threshold, the water has a greater than 50% chance of exceeding the *E. coli* standard. The final column contains the average of predicted model values using in situ turbidity data

Site	*E. coli* threshold	Model metrics				Model implementation	
		Accuracy	Sensitivity	Specificity	Turb where ***P***(***X***) = 0.50	In situ turb measurements	Avg ***P***(***X***)
Des Moines	235	85.2%	21.7%	97.8%	48.4	71,135	0.31
	2880	97.9%	6.8%	99.9%	191.7		0.11
Raccoon	235	82.3%	67.6%	90.3%	55.9	59,289	0.29
	2880	92.6%	34.7%	98.6%	270		0.03

### Recreational standard exceedance

In total, 71,135 and 59,289 in- itu turbidity measurements were recorded at the Des Moines and Raccoon sites, respectively (Table 3). This data allowed for a retrospective analysis exploring how frequently the sites exceeded the various standards. The resultant turbidity thresholds produced by the logistic models are plotted alongside the continuous turbidity datasets (Fig. 5). We then calculated the average probabilities estimated by the turbidity-based models for the entirety of the high-resolution datasets.

Des Moines’ turbidity record (71,135 measurements from 2019 to 2022) yielded an average probability of 0.31, suggesting the Class A1 standard was exceeded 31% of the time. Similarly, the Des Moines’ Class A2 model had an average probability of 0.11. Based on the Raccoon’s record (59,289 measurements from 2020 to 2022), average probabilities for the Class A1 and A2 thresholds were 0.29 and 0.03, respectively. As anticipated, average probabilities were larger for the Class A1 standard than for the Class A2, as each time *E. coli* levels rise above the Class A2 threshold, the Class A1 standard is also exceeded. Notably, average probabilities were also higher in the Des Moines than in the Raccoon for both standards.

## Discussion

### Relationship between turbidity, streamflow, and E. coli

The traditional relationships documented between turbidity, streamflow, and *E. coli* were noted in this study. In every case, *E. coli* was positively correlated with turbidity and streamflow at high levels of statistical significance (*p* < 0.001). Hydrologic processes, primarily surface runoff and storm sewer discharge, drive *E. coli* delivery to waterbodies in central Iowa (Brendel & Soupir, 2017). It was thus unsurprising that increased streamflow correlated with higher *E. coli* concentrations in this study. These same hydrologic processes can also result in erosion and sediment transport, and this likely accounted for the negligible model improvements gained when adding streamflow as a covariate alongside turbidity. Multicollinearity between streamflow and turbidity is common in river systems (Rasmussen et al., 2009), and this phenomenon is likely at play in the Des Moines and Raccoon Rivers. Since high streamflow often coincided with high turbidity, using both surrogates proved unnecessary in this study’s models.

It was notable that turbidity-based models performed better than their flow-based counterparts. In the case of Des Moines, this is likely related to Saylorville Lake. Due to this reservoir, streamflow levels in the Des Moines River are commonly linked with planned upstream discharge rates rather than recent precipitation events. Hydraulic impoundments often confound the typical transport pathways found in free-flowing systems, and Saylorville Lake has likely weakened the relationship between streamflow and *E. coli* widely found in larger Midwestern rivers (Fox et al., 2016). The relationship noted between streamflow and *E. coli* at Des Moines may reflect the influence of local tributaries rather than flows from Saylorville Lake. More broadly, however, turbidity may be a more suitable surrogate than streamflow in the region. In several instances, water clarity has been shown to better align with pathogen presence than streamflow (Hamilton & Luffman, 2009; Money et al., 2009).

Saylorville’s presence also resulted in significant differences in *E. coli* levels between the two sites, with concentrations generally being lower in Des Moines (Fig. 2). The removal of indicator bacteria in reservoir systems is well-documented (Reitter et al., 2021) and was observed in the Saylorville system when comparing upstream and downstream *E. coli* concentrations. The better model performance at the Raccoon site may have been related to this location having a more balanced *E. coli* dataset (i.e., a greater portion of samples above the EPA standards) and being subject to more free-flowing hydrologic conditions.

In any case, there may be several local factors driving *E. coli* levels not captured in these models. One probable contributor is wastewater treatment plants. While municipal point sources are required to ensure *E. coli* in their discharge meets regulatory thresholds, episodic releases may drive *E. coli* levels above the standards (Elmund et al., 1999). Such discharges are unlikely to be captured in streamflow or turbidity data, as point source discharges comprise small portions (< 20%) of the Des Moines and Raccoon Rivers’ overall flow. Other known sources include feces from wildlife and domestic animals (Guenther et al., 2011) and leaking septic tanks or municipal sewer systems (Guérineau et al., 2014). Local sources may only result in contamination on an event-specific basis, and episodic contamination events can result in disproportionate amounts of *E. coli* being released into source waters (McLellan et al., 2007). All these components contribute to the dynamics and variability of *E. coli*, thus making it difficult to quantify in the short term (Schilling et al., 2009). Adequately accounting for these localized variables will likely remain a persistent challenge in developing surrogacy models for *E. coli*.

### Model performance and viability

While statistically significant models predicting the exceedance of *E. coli* thresholds were created, their implementation warrants further consideration. Model accuracy values were high (82–98%), but this metric is somewhat misleading given the unbalanced nature of the *E. coli* datasets. For example, only 2.1% and 9.3% of samples exceeded the 2880 threshold in the Des Moines and Raccoon, respectively. Simply classifying every sample as being below 2880 MPN/100 mL would result in accuracy values > 90%. Adoption of these models is a policy decision and requires that decision-makers weigh the consequences of reporting false positives to those of false negatives.

The pseudo *R*^2^ and sensitivity metrics are better indicators of model performance when accounting for the unbalanced datasets. While it can be difficult to determine a model’s applicability solely based on its pseudo *R*^2^ (Heinzl et al., 2005), the values produced by this study’s models (0.19–0.38) do not indicate a strong goodness of fit. Perhaps more concerning are the sensitivities (6.8–68%). These low values indicate that the models are liable to produce many false negatives, i.e., predicting the water is below the *E. coli* standard when it is actually above it. Such behavior may raise concerns for agencies wishing to instill confidence in recreators by deploying these models. The Raccoon River Class A1 model was somewhat of an exception (sensitivity of 68%). However, this model also contained the lowest specificity (90%), suggesting that its performance metrics are more related to a larger percentage of training data exceeding the *E. coli* standard (35%) than improved goodness of fit. While accuracy and sensitivity values could be altered using a different probability threshold for classification (e.g., *P*(*X*) > 0.25 instead of *P*(*X*) > 0.5), further efforts should prioritize improved goodness of fit. Model implementation is ultimately at the discretion of local policymakers, but we recommend continued study before any real-time models are deployed.

### Future modeling efforts

Future modeling efforts could expand upon the methods used in this study. A variety of techniques could be utilized to further investigate whether using surrogates to predict the real-time exceedance of the *E. coli* thresholds has utility. First and foremost, our methods could be replicated in different locations with different datasets. Numerous agencies routinely collect turbidity and *E. coli* data, and streamflow is constantly measured across the US. However, it should be noted that our selection of the Des Moines and Raccoon sites was based, in part, on the large amounts of historical samples available (*n* > 4000). Given the large size of the existing dataset, adding new samples to this study’s training data seems unlikely to bolster model performance. Still, other locales may have alternative compositions of microorganism sources or transport pathways that result in clearer decision boundaries (Dorner et al., 2006).

Secondly, more sophisticated classification techniques could also be explored. Such supervised classification algorithms have shown promise in a wide variety of environmental applications (Deka, 2014; Gakii & Jepkoech, 2019; Modaresi & Araghinejad, 2014). For data used in this study, though, more complex modeling techniques are unlikely to improve model performance. The overlapping behavior of the two classes (Fig. 2) suggests that even more flexible decision boundaries may struggle with correctly classifying many of the data points. It may be the case that turbidity and streamflow alone are insufficient for classifying *E. coli* exceedance in the Des Moines and Raccoon Rivers. Certain classification methods also have the drawback of decreased interpretability, i.e., while models may perform well, it can be difficult to determine what factors they are using to produce their predictions (Lipton, 2018). Such models may make it difficult to infer the mechanisms that deliver bacteria to a given waterbody and may ultimately prove ineffective at guiding remediation efforts (Priyadarshini et al., 2022).

Finally, other researchers may wish to incorporate additional predictors into their training data. While turbidity and streamflow have demonstrated their usefulness as surrogates (Steffy & Shank, 2018), other parameters, which can be measured continuously in real-time, may help improve model performance. Water temperature, wind speed, and dissolved oxygen are among the other potential surrogates that may prove helpful (Baydaroğlu, 2025; Coffin et al., 2018). We recommend that future studies explore water temperature, in particular, since it has been found to greatly influence *E. coli* activity (Blaustein et al., 2013). We had initially hoped to incorporate temperature among our suite of predictors, but discrepancies and inconsistencies in temperature records at both sites dissuaded us from using this analyte. Still, because of its well-established link with *E. coli* and the simplicity of its measurement, temperature is likely the best candidate for an additional predictor. It should be noted, however, that while the inclusion of additional parameters may improve model performance, it also increases the likelihood of model failure. Models requiring multiple in situ datasets risk disruption by a single incidence of sensor fouling (Anderson et al., 2024). Numerous sensors at a site increase financial and logistical burdens due to the added cost and maintenance associated with ensuring all devices are operating correctly (Razavi et al., 2012).

There may be a useful parallel between our modeling framework and the development of nowcasting models used to predict the occurrence of harmful algal blooms (HABs) (Francy et al., 2019). Toxins produced by HABs are another threat to recreators and have been documented in the Des Moines and Raccoon Rivers (Backer et al., 2015). HABs are notoriously difficult to predict, but some efforts have shown promise in warning stakeholders of their potential occurrence using various in situ water quality and climatic parameters (Baydaroğlu, 2025; Ralston & Moore, 2020). Considerable resources have been devoted to creating these models—far beyond those of this study—but the techniques that have been employed may help guide *E. coli* modeling efforts. Furthermore, it may be advisable to incorporate *E. coli* prediction models in areas where HAB nowcasting is already being deployed.

### Surrogates as indicators of long-term recreational risk

This study’s models may have a secondary use case in long-term assessments of recreational risk. The collection of in situ turbidity data enabled a new method for quantifying a waterway’s long-term tendency to exceed recreational standards. Assessing microbial indicator levels in a waterbody has remained challenging, given *E. coli*’s dynamic nature and ability to span multiple orders of magnitude. Many historical monitoring programs collect discrete grab samples on a weekly or monthly basis. Such protocols can result in large uncertainties surrounding many analytes due to temporal gaps between sample collection (Tate et al., 1999).

Deploying a surrogacy model allows for repeated measurements at lengths and resolutions typically impractical with grab sampling (Jones et al., 2011). Additionally, many time-integrated passive sampling techniques are also unavailable for *E. coli* due to microbe perishability (Hayes & Gagnon, 2024). Therefore, repeated predictions of threshold exceedance probability may prove more beneficial in describing a waterbody’s suitability for recreation than historical estimates based on intermittent sampling.

Further study is needed to quantify the accuracy of these techniques, but there are ample opportunities for models to be deployed in this context. While we advise against collecting new water quality data solely for constructing similar surrogacy models, many sites have historical datasets containing turbidity and *E. coli* that can be leveraged. Likewise, a handful of these sites also contain turbidimeters. In such cases, model implementation, as described in this study, could be conducted without incurring large additional expenses. These models could be retroactively applied to gauge high-resolution exceedance of thresholds over the course of months or years. At sites without turbidimeters, agencies may wish to install devices for this expressed purpose after exploring the local viability of surrogacy models. This method may prove valuable in helping prioritize improvement efforts by identifying waterbodies most prone to exceeding recreational standards.

## Conclusions

This study explored the potential of turbidity and streamflow to predict the exceedance of aquatic recreational standards in the Des Moines and Raccoon Rivers. Specifically, logistic models were used to estimate the probability of exceeding *E. coli* levels of 235 and 2880 MPN/100 mL. Turbidimeters were also deployed to collect high-resolution observations and aid with model implementation. Four models were ultimately selected—one for both rivers and standards—that used turbidity as the lone predictor. Including streamflow did not bolster model performance.

While these models were workable, their effectiveness was mixed. We caution against collecting new data solely to construct these sorts of models, as our setup utilized an extensive historical dataset and still exhibited weak performances. In these two rivers analyzed, indicator bacteria concentrations are likely influenced by local upstream conditions that are only partially correlated with water clarity and hydrology. Future studies could include additional surrogates in modeling efforts and explore more sophisticated classification algorithms.

The collected in situ turbidity data enabled novel estimates of recreational threshold exceedances, and these may be more thorough than traditional assessments conducted using discrete samples. Agencies may wish to replicate our methods in similar scenarios, i.e., where large amounts of water quality data have previously been collected. Such efforts may yield improved descriptions of a waterbody’s recreational suitability across specific periods of interest.

## Supplementary information

Below is the link to the electronic supplementary material.

ESM 1 (ZIP 11.0 MB)

## Data Availability

All data used in this study are publicly available and have also been provided in the Supplemental Materials. *E. col*i and turbidity sampling data are available via personal communication with local operators at DMWW. Streamflow data can be retrieved through the USGS National Water Information System (https://waterdata.usgs.gov/nwis). In situ turbidity data can be retrieved through the IWQIS database (https://iwqis.iowawis.org/).
