# Supplementary material for: Turbidity and streamflow as real-time indicators of microbial risk for aquatic recreators
Source: Environ Monit Assess. 2026 Apr 28;198(5):513. doi: 10.1007/s10661-026-15370-6 (PMC13124811; doi:10.1007/s10661-026-15370-6)
Supplement: Supplementary file 1 — (ZIP 11.0 MB) [file 10661_2026_15370_MOESM1_ESM.zip › supplemental/model parameters and metrics/Streamflow/Des Moines_235_Flow.pdf]

Model Details [site: Des Moines], [E. coli threshold: 235], [Predictor(s): Flow]

Model Specifications and Performance Metrics			
Dep. Variable:	235 Ecoli	No. Observations:	4188
Model:	Logit	Df Residuals:	4186
Method:	MLE	Df Model:	1
Date:	18 Jan 2025	Pseudo R-squ.:	0.02761
Time:	9:12:07	Log-Likelihood:	-1834.9
converged:	True	LL-Null:	-1887
Covariance Type:	nonrobust	LLR p-value:	1.84E-24

Model Coefficients and P-Values						
	coef	std err	z	P>|z|	[0.025	0.975]
Intercept	-4.2086	0.274	-15.356	0	-4.746	-3.671
Flow_log	0.3265	0.033	9.825	0	0.261	0.392
