# Supplementary material for: Turbidity and streamflow as real-time indicators of microbial risk for aquatic recreators
Source: Environ Monit Assess. 2026 Apr 28;198(5):513. doi: 10.1007/s10661-026-15370-6 (PMC13124811; doi:10.1007/s10661-026-15370-6)
Supplement: Supplementary file 1 — (ZIP 11.0 MB) [file 10661_2026_15370_MOESM1_ESM.zip › supplemental/model parameters and metrics/Streamflow/Raccoon_235_Flow.pdf]

Model Details [site: Raccoon], [E. coli threshold: 235], [Predictor(s): Flow]

Model Specifications and Performance Metrics			
Dep. Variable:	235 Ecoli	No. Observations:	4175
Model:	Logit	Df Residuals:	4173
Method:	MLE	Df Model:	1
Date:	18 Jan 2025	Pseudo R-squ.:	0.154
Time:	9:12:16	Log-Likelihood:	-2294
converged:	True	LL-Null:	-2711.5
Covariance Type:	nonrobust	LLR p-value:	1.33E-183

Model Coefficients and P-Values						
	coef	std err	z	P>|z|	[0.025	0.975]
Intercept	-6.4076	0.244	-26.308	0	-6.885	-5.93
Flow_log	0.7871	0.032	24.708	0	0.725	0.85
