# Supplementary material for: Turbidity and streamflow as real-time indicators of microbial risk for aquatic recreators
Source: Environ Monit Assess. 2026 Apr 28;198(5):513. doi: 10.1007/s10661-026-15370-6 (PMC13124811; doi:10.1007/s10661-026-15370-6)
Supplement: Supplementary file 1 — (ZIP 11.0 MB) [file 10661_2026_15370_MOESM1_ESM.zip › supplemental/model parameters and metrics/Streamflow/Raccoon_2880_Flow.pdf]

Model Details [site: Raccoon], [E. coli threshold: 2880], [Predictor(s): Flow]

Model Specifications and Performance Metrics			
Dep. Variable:	2880 Ecoli	No. Observations:	4175
Model:	Logit	Df Residuals:	4173
Method:	MLE	Df Model:	1
Date:	18 Jan 2025	Pseudo R-squ.:	0.1409
Time:	9:12:37	Log-Likelihood:	-1111.2
converged:	True	LL-Null:	-1293.5
Covariance Type:	nonrobust	LLR p-value:	2.94E-81

Model Coefficients and P-Values						
	coef	std err	z	P>|z|	[0.025	0.975]
Intercept	-9.0844	0.441	-20.583	0	-9.949	-8.219
Flow_log	0.8784	0.053	16.476	0	0.774	0.983
