# Supplementary material for: Turbidity and streamflow as real-time indicators of microbial risk for aquatic recreators
Source: Environ Monit Assess. 2026 Apr 28;198(5):513. doi: 10.1007/s10661-026-15370-6 (PMC13124811; doi:10.1007/s10661-026-15370-6)
Supplement: Supplementary file 1 — (ZIP 11.0 MB) [file 10661_2026_15370_MOESM1_ESM.zip › supplemental/model parameters and metrics/Turbidity/Raccoon_235_Turbidity.pdf]

Model Details [site: Raccoon], [E. coli threshold: 235], [Predictor(s): Turbidity]

Model Specifications and Performance Metrics			
Dep. Variable:	235 Ecoli	No. Observations:	4170
Model:	Logit	Df Residuals:	4168
Method:	MLE	Df Model:	1
Date:	18 Jan 2025	Pseudo R-squ.:	0.3498
Time:	9:12:11	Log-Likelihood:	-1760.3
converged:	True	LL-Null:	-2707.5
Covariance Type:	nonrobust	LLR p-value:	0.00E+00

Model Coefficients and P-Values						
	coef	std err	z	P>|z|	[0.025	0.975]
Intercept	-6.5267	0.203	-32.153	0	-6.925	-6.129
Flow_log	1.6219	0.053	30.818	0	1.519	1.725
