# Supplementary material for: Turbidity and streamflow as real-time indicators of microbial risk for aquatic recreators
Source: Environ Monit Assess. 2026 Apr 28;198(5):513. doi: 10.1007/s10661-026-15370-6 (PMC13124811; doi:10.1007/s10661-026-15370-6)
Supplement: Supplementary file 1 — (ZIP 11.0 MB) [file 10661_2026_15370_MOESM1_ESM.zip › supplemental/model parameters and metrics/Turbidity and Streamflow/Des Moines_2880_Turbidity and Streamflow.pdf]

Model Details [site: Des Moines], [E. coli threshold: 2880], [Predictor(s): Turbidity & Flow]

Model Specifications and Performance Metrics			
Dep. Variable:	2880 Ecoli	No. Observations:	4185
Model:	Logit	Df Residuals:	4182
Method:	MLE	Df Model:	1
Date:	18 Jan 2025	Pseudo R-squ.:	0.2294
Time:	9:12:31	Log-Likelihood:	-328.98
converged:	True	LL-Null:	-426.92
Covariance Type:	nonrobust	LLR p-value:	2.93E-43

Model Coefficients and P-Values						
	coef	std err	z	P>|z|	[0.025	0.975]
Intercept	-9.0183	0.845	-10.676	0	-10.67	-7.363
Turb_log	1.8926	0.155	12.221	0	1.589	2.196
Flow_log	-0.099	0.107	-0.929	0.353	-0.308	0.11
