# Supplementary material for: Turbidity and streamflow as real-time indicators of microbial risk for aquatic recreators
Source: Environ Monit Assess. 2026 Apr 28;198(5):513. doi: 10.1007/s10661-026-15370-6 (PMC13124811; doi:10.1007/s10661-026-15370-6)
Supplement: Supplementary file 1 — (ZIP 11.0 MB) [file 10661_2026_15370_MOESM1_ESM.zip › supplemental/model parameters and metrics/Turbidity and Streamflow/Raccoon_2880_Turbidity and Streamflow.pdf]

Model Details [site: Raccoon], [E. coli threshold: 2880], [Predictor(s): Turbidity & Flow]

Model Specifications and Performance Metrics			
Dep. Variable:	2880 Ecoli	No. Observations:	4170
Model:	Logit	Df Residuals:	4167
Method:	MLE	Df Model:	1
Date:	18 Jan 2025	Pseudo R-squ.:	0.3895
Time:	9:12:41	Log-Likelihood:	-789.33
converged:	True	LL-Null:	-1293
Covariance Type:	nonrobust	LLR p-value:	1.80E-219

Model Coefficients and P-Values						
	coef	std err	z	P>|z|	[0.025	0.975]
Intercept	-8.505	0.462	-18.415	0	-9.41	-7.6
Turb_log	1.9109	0.094	20.233	0	1.726	2.096
Flow_log	-0.2482	0.068	-3.673	0	-0.381	-0.116
