# Supplementary figures and images for: Turbidity and streamflow as real-time indicators of microbial risk for aquatic recreators
Source: Environ Monit Assess. 2026 Apr 28;198(5):513. doi: 10.1007/s10661-026-15370-6 (PMC13124811; doi:10.1007/s10661-026-15370-6)

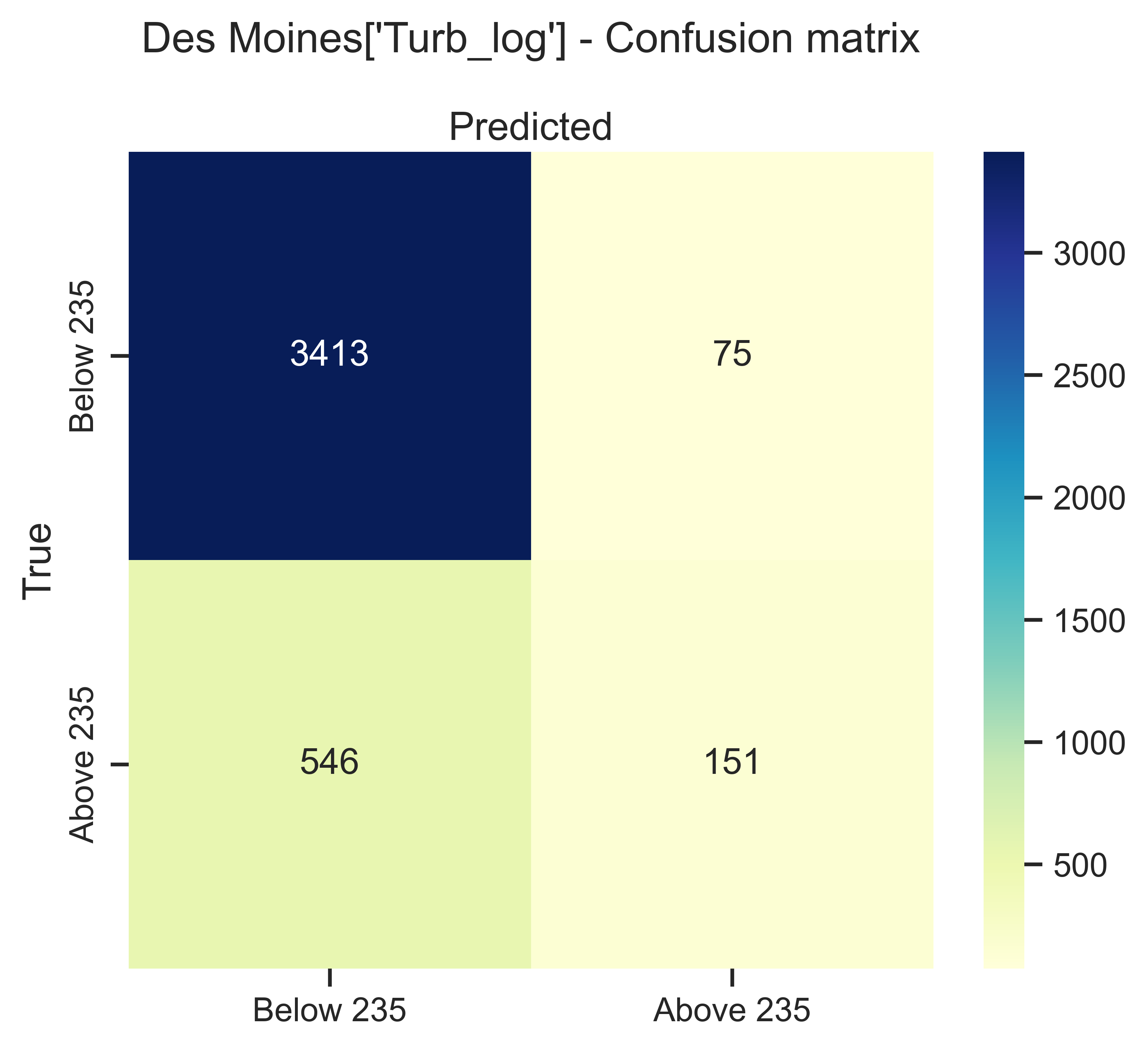

Supplement: Supplementary file 1 — (ZIP 11.0 MB) [file 10661_2026_15370_MOESM1_ESM.zip › supplemental/confusion matrices/Des Moines235VsTurb_log_ConMatrixLog.png]

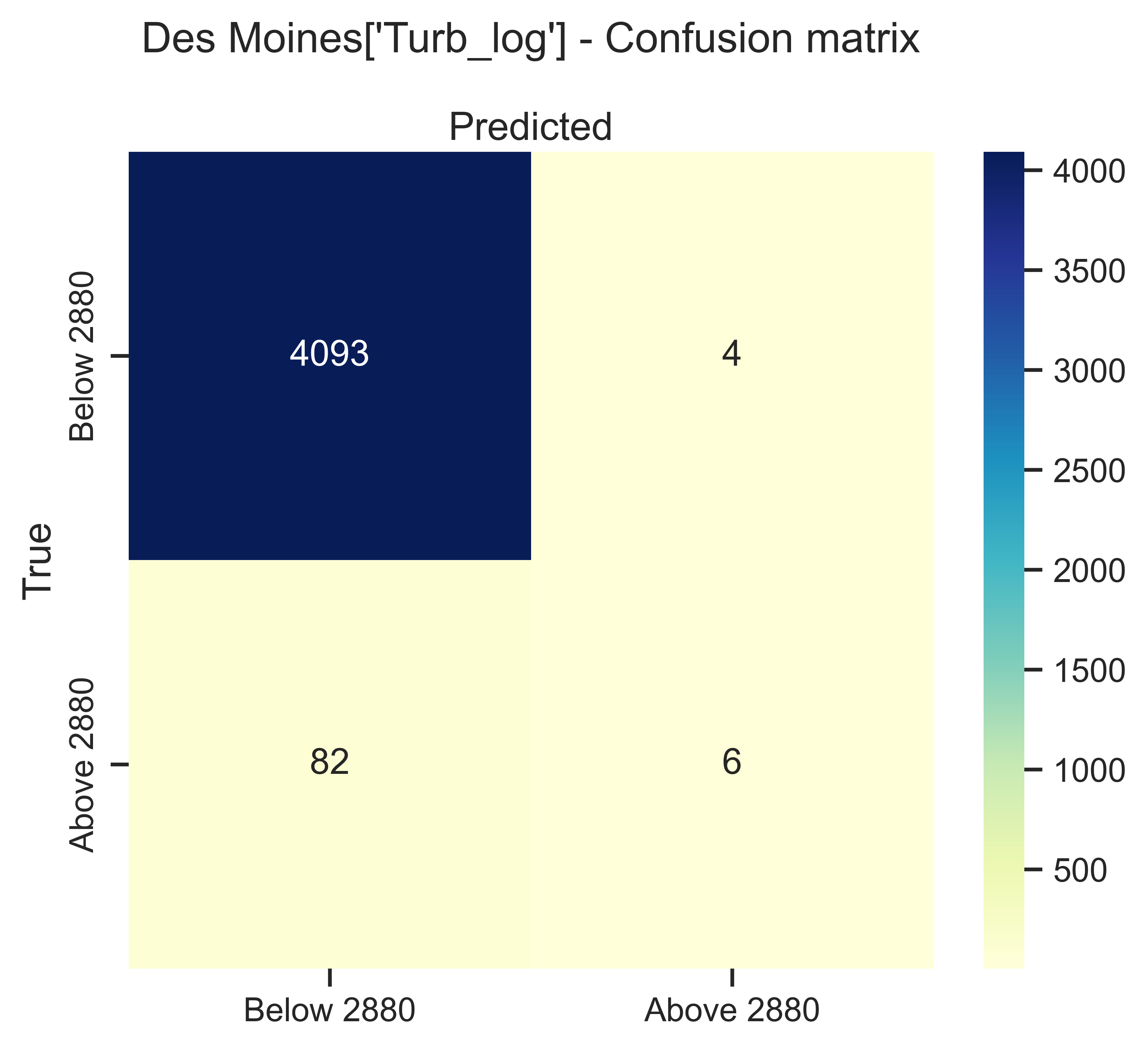

Supplement: Supplementary file 1 — (ZIP 11.0 MB) [file 10661_2026_15370_MOESM1_ESM.zip › supplemental/confusion matrices/Des Moines2880VsTurb_log_ConMatrixLog.png]

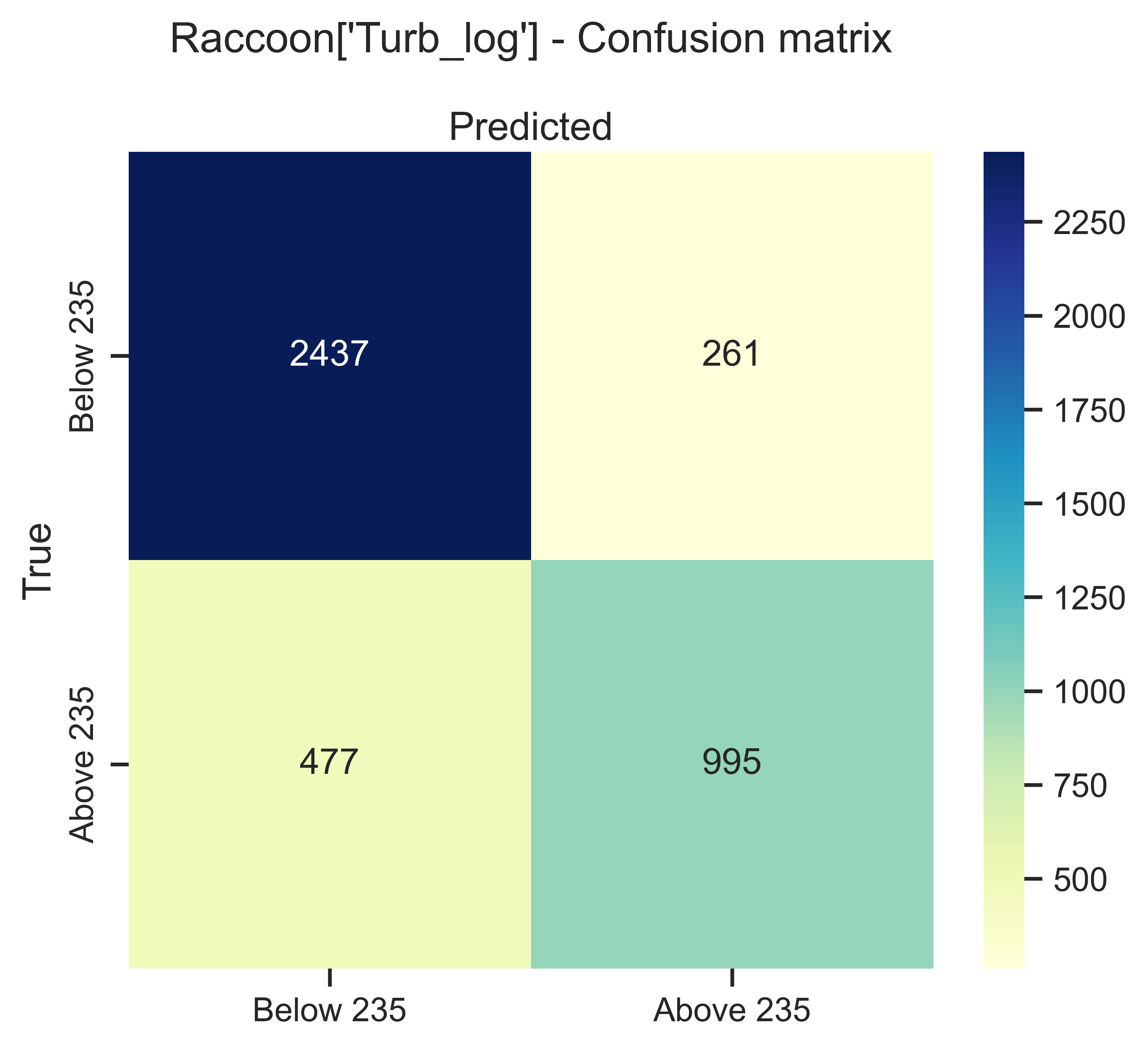

Supplement: Supplementary file 1 — (ZIP 11.0 MB) [file 10661_2026_15370_MOESM1_ESM.zip › supplemental/confusion matrices/Raccoon235VsTurb_log_ConMatrixLog.png]

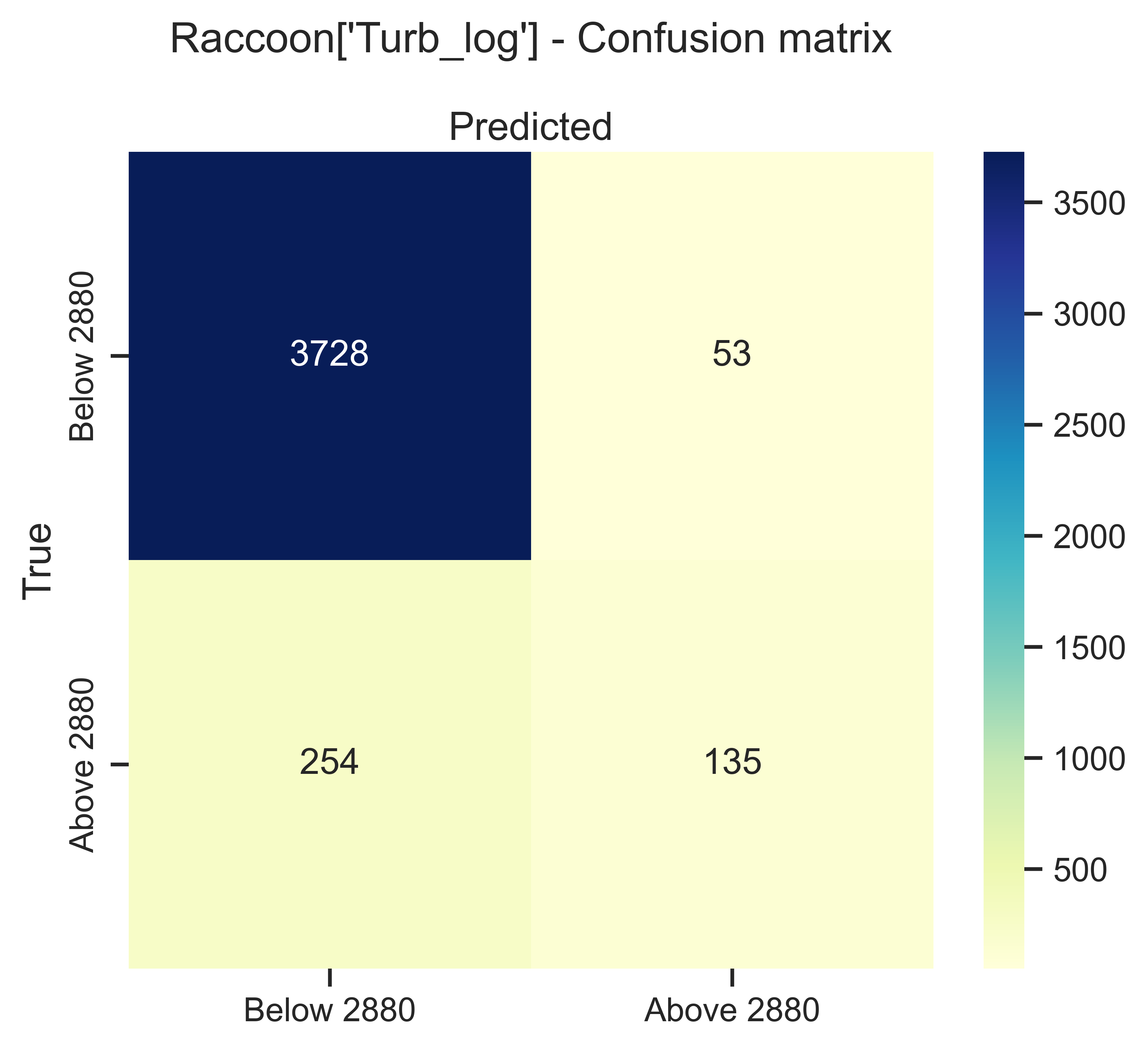

Supplement: Supplementary file 1 — (ZIP 11.0 MB) [file 10661_2026_15370_MOESM1_ESM.zip › supplemental/confusion matrices/Raccoon2880VsTurb_log_ConMatrixLog.png]

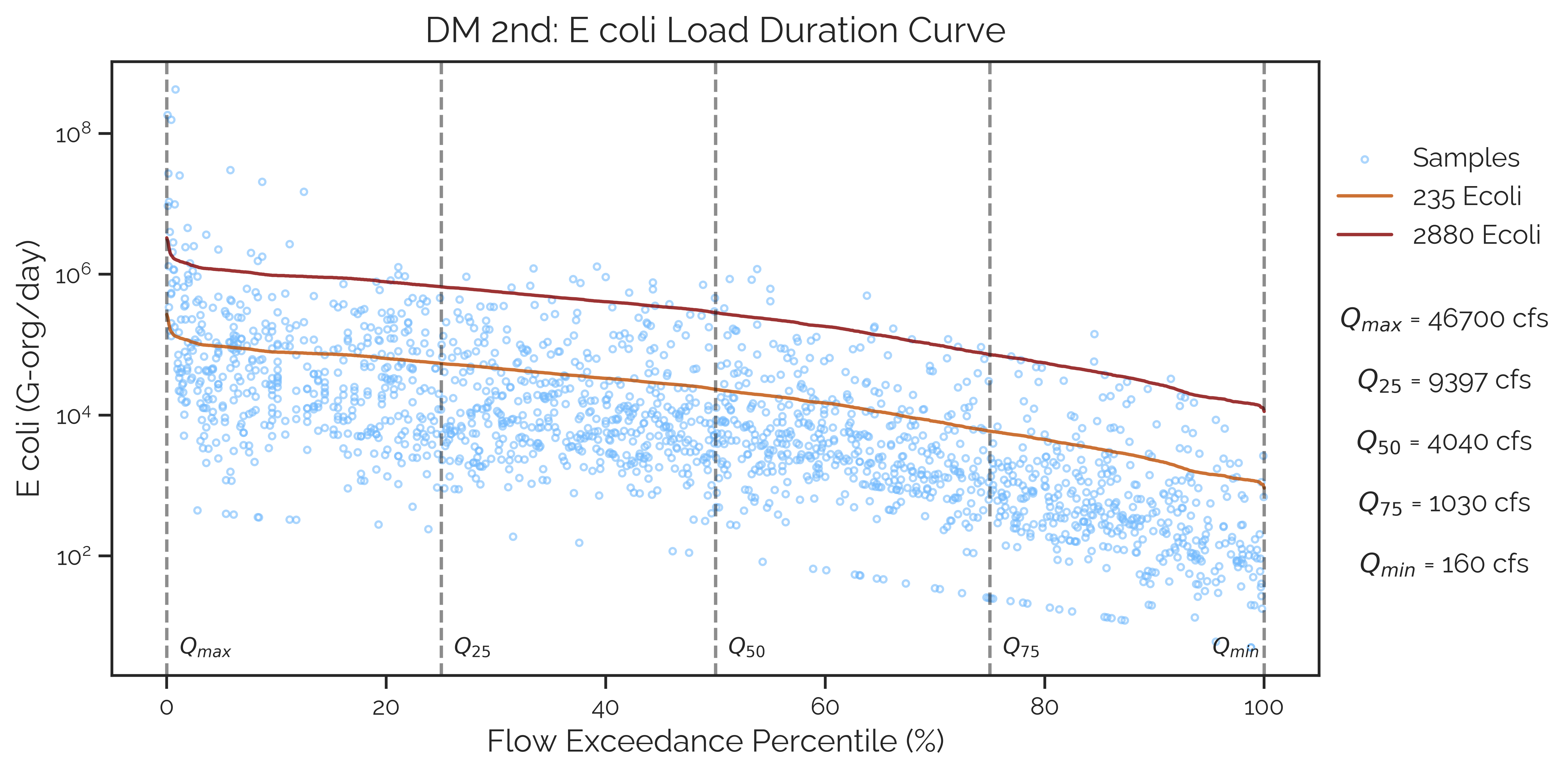

Supplement: Supplementary file 1 — (ZIP 11.0 MB) [file 10661_2026_15370_MOESM1_ESM.zip › supplemental/load duration curves/DM_LoadDuration.png]

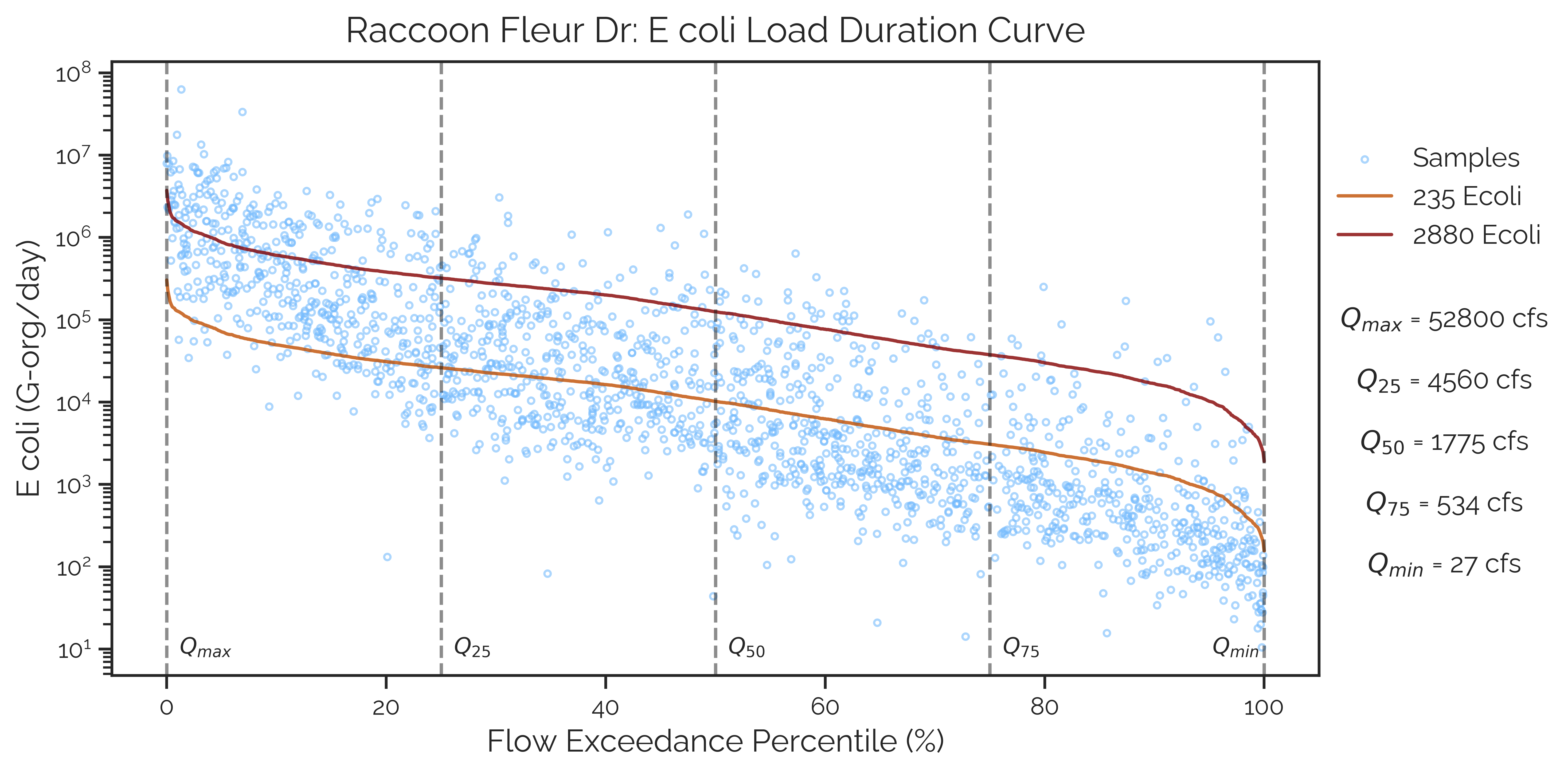

Supplement: Supplementary file 1 — (ZIP 11.0 MB) [file 10661_2026_15370_MOESM1_ESM.zip › supplemental/load duration curves/Raccoon_LoadDuration.png]
